# Tracking Aromatic Volatile Biomarkers Through Coffee Bean Postharvest Stages

**DOI:** 10.3390/molecules31050853

**Published:** 2026-03-04

**Authors:** Alexa J. Pajuelo-Muñoz, Ilse S. Cayo-Colca, Carlos Granda-Wong, Renan Campos Chisté, Efraín M. Castro-Alayo, César R. Balcázar-Zumaeta

**Affiliations:** 1 Instituto de Investigación, Innovación y Desarrollo para el Sector Agrario y Agroindustrial (IIDAA), Facultad de Ingeniería y Ciencias Agrarias, Universidad Nacional Toribio Rodríguez de Mendoza de Amazonas, Calle Higos Urco 342-350-356, Chachapoyas 01001, Peru; 7255974661@untrm.edu.pe (A.J.P.-M.); efrain.castro@untrm.edu.pe (E.M.C.-A.); 2 Facultad de Ingeniería Zootecnista, Biotecnología, Agronegocios y Ciencia de Datos, Universidad Nacional Toribio Rodríguez de Mendoza de Amazonas, Calle Higos Urco 342-350-356, Chachapoyas 01001, Peru; icayo.fizab@untrm.edu.pe; 3 Department of Plant Pathology and Agricultural Engineering, Universidad Nacional de Piura, Campus Universitario, Urb. Miraflores s/n, Piura 20008, Peru; cgrandaw@unp.edu.pe; 4 Faculdade de Farmácia, Campus Belo Horizonte, Universidade Federal de Minas Gerais, Belo Horizonte 31270-901, Brazil; rcchiste@ufmg.br

**Keywords:** alcohols, biomarker, coffee, fermentation, roasted, volatile compounds

## Abstract

This review synthesizes recent evidence on the generation and behavior of volatile biomarkers throughout the main postharvest stages of coffee, highlighting their potential for technological standardization. During harvest, aldehydes, furans, and lactones reflect ripeness and the presence of physiological defects, thereby influencing the formation of other volatile groups in subsequent stages. During pulping and fermentation, the metabolism of yeasts and lactic and acetic acid bacteria produces alcohols, acids, and esters (such as 2-phenylethanol, ethyl acetate, and methyl phenylacetate), which function as biomarkers of proper mucilage management and a balanced initial fermentation. In drying, the evolution of aldehydes derived from lipid oxidation and the retention of aromatic esters provide insights into dehydration kinetics and the stability of green coffee against oxidation. Finally, during roasting, volatile pyrazines, furans, thiols, and phenols integrate the entire postharvest history of the bean and enable inferences about roast degree, thermal overexposure, and final aroma development. Overall, the volatile biomarkers described here provide a robust chemical basis for objective monitoring of the postharvest process and the differentiation of coffee lots, although further studies are needed to define critical ranges by origin and processing system, standardize analytical methodologies, and quantitatively link these compounds to commercial quality parameters.

## 1. Introduction

Coffee is an important agricultural commodity worldwide and a widely consumed beverage, owing to its chemical composition and its socioeconomic importance. The *Rubiaceae* species *Coffea arabica* L. and *Coffea canephora* Pierre ex A. Froehner are the most widely cultivated and represent an essential source of income for tropical and subtropical regions [1,2]. The final quality of coffee depends on several factors, including the chemical composition of the bean, cultivation conditions, and the transformation processes that occur during postharvest handling [3,4]. In particular, the aromatic profile determined by the presence and balance of volatile compounds constitutes one of the most influential sensory attributes in the acceptance of the final product [5,6,7].

More than eight hundred volatile compounds have been identified in coffee beans, belonging to groups such as aldehydes, alcohols, acids, esters, ketones, furans, pyrazines, and phenols, although only a fraction of them contributes significantly to the perceived aroma [8]. Recent studies confirm that this chemical diversity derives from well-defined metabolic pathways rather than random processes, resulting from enzymatic and non-enzymatic transformations that occur throughout postharvest processing [1,5]. Among the most relevant pathways are microbial fermentation, the oxidation of unsaturated lipids, and the thermal Maillard and Strecker reactions during roasting, which are responsible for generating aldehydes, higher alcohols, acids, and pyrazines that impart floral, fruity, or roasted notes to the bean [6,9]. This compositional complexity makes the volatile profile of coffee a sensitive indicator of processing conditions and a fundamental starting point for the identification of quality biomarkers.

**Figure 1:**
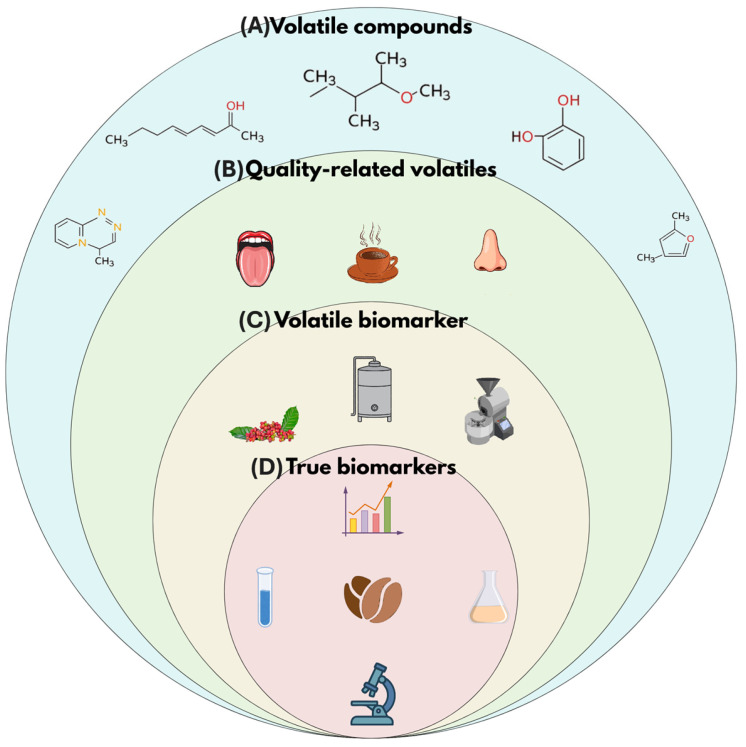
Hierarchical classification framework for volatile compounds in coffee postharvest processing. The classification ranges from general chemical detection to validation as a process control tool. (**A**) Volatile compound: Total volatile fraction detected without specific functional relevance. (**B**) Quality-related volatile: Compounds associated with sensory descriptors or defects. (**C**) Volatile biomarker: Candidate signals linked to specific processing stages (e.g., fermentation, roasting). (**D**) True biomarker: Validated signals demonstrating high reproducibility, discriminative capacity, and documented utility for decision-making.

A volatile compound is defined as any molecule detected within the volatile fraction of a given matrix using a specified analytical approach. Within this, we differentiate quality-related volatiles, referring to compounds—or compound combinations— whose presence or abundance has been associated in the literature with desirable sensory attributes or defects, yet without requiring stage specificity or independent discriminative capability. In contrast, the term volatile biomarker is reserved for compounds or compound patterns for which cumulative evidence supports their role as consistent indicators of a defined stage or condition. A biomarker is understood as any compound whose presence, concentration, or proportion reflects a physiological condition, a process stage, or a quality state of the bean [5,10]. A conceptual distinction is further established between candidate biomarkers and true biomarkers: candidate biomarkers are supported by recurrent reports but remain constrained by limited validation, whereas true biomarkers demonstrate inter-study reproducibility, discriminatory robustness across closely related stages or conditions, and documented analytical performance suitable for monitoring or process control applications (Figure 1).

On this basis, the concept of a volatile biomarker during fermentation acquires relevance in coffee research, as it enables the linkage between aromatic composition and the biochemical dynamics of postharvest processing. In coffee, volatile biomarkers function as chemical tracers that allow specific molecules to be associated with particular processing phases or with sensory deviations attributable to unbalanced fermentations [11,12]. For example, elevated levels of acetic acid and ethanol are associated with fermentations in which microbial activity is not adequately controlled, leading to sensory defects such as vinegary or sharp notes. In contrast, higher concentrations of 2-phenylethanol, ethyl acetate, or isoamyl acetate are linked to fermentations conducted under well-regulated conditions, which produce fruity or floral aromatic profiles [13,14,15]. In this context, the identification of volatile biomarkers represents a powerful tool for correlating microbial metabolism with final coffee quality and for establishing objective indicators for postharvest standardization.

**Figure 2:**
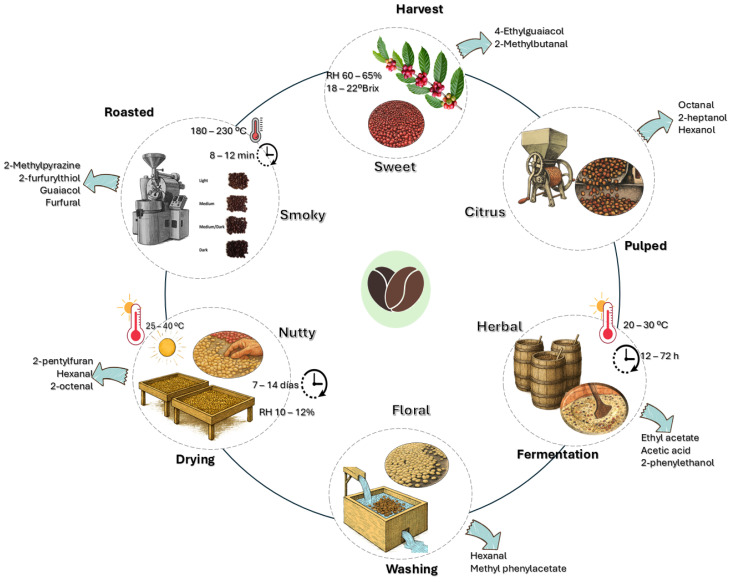
Postharvest stages of coffee: processing conditions, candidate volatile markers, and associated sensory attributes. The scheme outlines the sequence of unit operations involved after harvesting, including cherry selection, pulping, fermentation, washing, drying, and roasting. Each stage influences bean quality by affecting the biochemical and microbiological dynamics that determine the final sensory attributes.

Each postharvest stage, from harvesting to roasting, involves a complex network of interdependent chemical and biochemical transformations that determine the set of volatile compounds formed. During harvesting, enzymatic and oxidative reactions predominate in the plant tissue of the bean, promoting the formation of short-chain aldehydes and alcohols derived from the degradation of lipids and phenolic compounds [16,17]. During pulping and fermentation, the microbial activity of yeasts and lactic acid bacteria drives the production of alcohols, organic acids, and esters through the conversion of sugars and amino acids, generating compounds such as ethanol, acetic acid, ethyl acetate, and 2-phenylethanol [14,18,19]. In the drying and roasting stages, thermal conditions induce Maillard and Strecker reactions, as well as lipid oxidation, giving rise to the formation of aldehydes, pyrazines, furans, and phenols responsible for the characteristic aroma of coffee [20,21,22,23]. An integrated understanding of these mechanisms allows not only the explanation of the origin of volatile compounds but also the interpretation of their function as quality indicators and their potential application as standardization biomarkers in postharvest processes [5]. These stages and their main critical points for volatile compound formation are summarized schematically in Figure 2.

In the coffee industry, volatile biomarkers are increasingly employed as supportive chemical variables for quality control and, in some cases, for process adjustment, as they provide objective measurements of changes that sensory panels cannot always assess or with equivalent reproducibility. In practice, their role is better understood in terms of operational decision-making. In commercial products and blends, the characterization of aroma-active compounds using established methods (e.g., GC-O/GC-MS, AEDA) enables the identification of chemical signals underlying sensory differences between lots or formulations. Such evidence extends beyond mere aroma description; it supports profile standardization and justifies adjustments in raw material selection or blending strategies when sensory perception shifts. In this way, sensory differences are translated into verifiable chemical drivers, strengthening quality assurance decisions at the industrial scale [24].

Conversely, in green coffee, volatile analysis has been applied as a screening tool for defect detection prior to investment in subsequent processing stages. Elevated levels of volatile carbonyl compounds in defective beans are particularly relevant, as these molecules function as early indicators of deterioration or oxidative damage. Their detection enables the classification of lots with a higher risk of aromatic quality loss, even before thermal transformation. Accordingly, the identification of such compounds supports lot segregation, adjustment of storage conditions, and prioritization of postharvest processing strategies, thereby reducing the probability of downstream quality depreciation [25]. Finally, during roasting—a stage in which adjustments in time and temperature are directly reflected in the final profile—recent studies have shown that the evolution of volatile families correlates with sensory attributes. Monitoring changes in compound groups associated with key aroma notes allows modifications in roasting parameters to be linked to measurable variations in both the volatile profile and sensory perception. From an industrial perspective, this supports the use of volatile biomarkers as verification variables. When a batch deviates from its expected roasting pattern, the volatile profile can help determine whether the variation originates from the raw material or from roaster configuration, while also providing a chemical basis to document the corrective adjustments applied to recover the target profile [20,26]. Overall, the role of volatile compounds in the coffee industry is not to replace sensory evaluation, but to complement it with chemically comparable criteria across three primary applications: (i) explanation and profile standardization in commercial products, (ii) early defect detection in green coffee, and (iii) verification and adjustment of roasting conditions to ensure final profile reproducibility.

## 2. Biosynthesis Routes and Abiotic Mechanisms of Aroma Compound Formation During Coffee Postharvest Processing

The volatile fraction of coffee can be interpreted within two principal formation frameworks: biosynthetic routes, associated with biological activity of plant tissues and microbial metabolism during processing, and abiotic routes, linked to oxidation and non-enzymatic chemical reactions governed by environmental conditions and, particularly, by roasting. This distinction is useful because the same volatile compound may arise at different stages through distinct mechanisms; therefore, its interpretation as an indicator of stage or processing practice depends on the compatibility between the proposed formation mechanism, the matrix, and the prevailing process conditions (oxygen availability, moisture, time, and temperature) [27,28].

### 2.1. Biosynthetic Pathways

**Figure 3:**
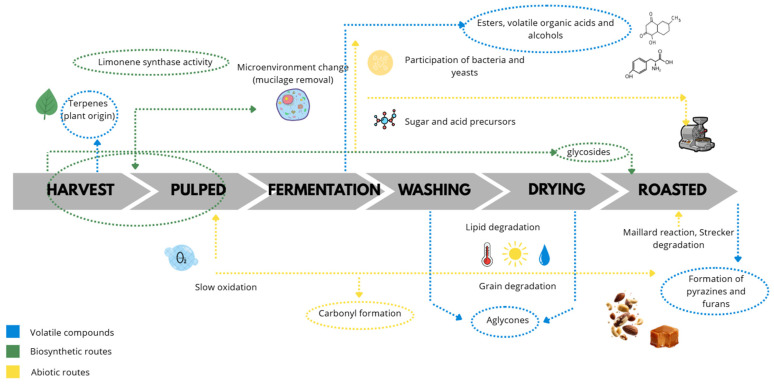
Dynamic map of volatile fraction evolution during coffee postharvest processing. Two formation frameworks are distinguished: (i) biosynthetic routes, including endogenous grain metabolism (e.g., terpenes) and microbial metabolism during fermentation (alcohols, organic acids, and esters); and (ii) abiotic routes, dominated by lipid oxidation during storage (carbonyl compounds) and by thermal reactions such as Maillard and Strecker pathways during roasting (pyrazines, furans, and sulfur-containing compounds). Arrows indicate precursor flow and the transition from biologically derived signals to thermally driven process markers.

From harvest onward, the bean inherently contains its own chemical fraction *per se* (Figure 3). At this initial stage, the terpenes present do not originate from fermentation or roasting, but from endogenous biosynthesis within the plant tissue, which may vary according to genotype. Therefore, these compounds represent an intrinsic chemical signature of the bean. In *Coffea arabica*, functional monoterpene synthases have been identified, including limonene synthase, confirming that part of the terpene fraction is directly plant-derived and independent of subsequent processing transformations [29]. In line with this biosynthetic framework, certain terpenes not only reflect process-related variations but may also act as markers of the biological origin and the inherent aromatic potential of the bean. In this regard, studies integrating sensory analysis and volatile compound profiling have demonstrated an association between the expression of genes involved in terpene biosynthesis, the higher abundance of limonene —identified as the predominant terpene in the profile— and the perception of citrus notes in the beverage, reinforcing their interpretation as indicators of the intrinsic contribution of the plant material [30]. Although the relative contribution of each family may vary depending on cultivar and fruit maturity stage, this biosynthetic framework is also consistent with the contribution of carotenoid-derived compounds, which depend on tissue status and subsequent biochemical transformations. Following depulping, the microenvironment of the bean is altered due to the removal of the exocarp and mucilage; this modifies the availability of fermentable substrates and defines the conditions under which fermentation develops. During fermentation, the dominant biosynthetic input is microbial and is mainly expressed through alcohols, acids, and esters generated by the conversion of sugars and amino acids. These chemical families are particularly informative because they depend on metabolic activity and respond to changes in pH, oxygen availability, and temperature [27,31]. Following washing, the intensity of these signals may decrease in the superficial fraction due to the removal of mucilage and the associated aqueous phase. Therefore, when comparing samples before and after washing, the analyzed matrix must be strictly defined to prevent confounding chemical transformations with changes arising from compartmental redistribution.

A key mechanism linking the green bean to later processing stages involves the transformation of conjugated precursors into volatile aroma compounds. Glycosylated volatiles constitute a precursor pool capable of releasing aromatic aglycones. In coffee, hydrolysis of green bean glycosides during roasting has been reported, along with the formation of newly generated glycosides within the roasted matrix, indicating that this fraction actively participates in aroma development [32]. This mechanism helps explain why certain aroma compounds increase during roasting, even when their free fraction in the green bean is low [33].

### 2.2. Abiotic Mechanism

Aroma formation throughout the postharvest process indicates that a considerable proportion of the volatiles present in the green bean does not persist directly after roasting, suggesting that the differences observed between stages are not explained solely by the initial presence of free compounds, but also by the transformation of precursors and changes in reaction conditions [28]. In this context, relevant abiotic pathways may be initiated prior to roasting and evolve according to handling practices and environmental conditions (Figure 3). From harvesting and early handling stages, exposure to oxygen, temperature fluctuations, and mechanical damage may trigger slow oxidative processes. During drying and storage, these transformations become dominant sources of changes in carbonyl compounds and other classes associated with green coffee aging. Such reactions consume susceptible substrates and alter the chemical stability of the bean, thereby conditioning the profile expressed after roasting. Consistently, a comparative study on green and roasted coffee demonstrated that the volatile profile can discriminate quality, roast level, origin, and defects, supporting the notion that signals modulated prior to roasting may persist as relevant differences and influence the final outcome [34].

During roasting, abiotic formation dominates through thermal reactions; the Maillard reaction and Strecker degradation generate multiple classes of odorants, notably pyrazines associated with roasted and nutty notes, and furanic compounds related to caramel-like attributes. In addition, the final profile includes sulfur-containing compounds with high odor potency, whose sensory contribution may be disproportionate to their concentration. Therefore, many roasting-derived volatiles are better interpreted as integrators of process time and temperature, as well as precursor availability (reducing sugars and amino acids), rather than as direct indicators of fermentation, unless a precursor–product linkage is experimentally demonstrated. One example is the conversion of furfuryl alcohol to furfurylthiol, verified through bean-level experiments involving precursor addition, stable isotope dilution analysis, and labeled precursor tracing, where an increase in furfurylthiol and isotopic label transfer to the product were observed after roasting [35]. Overall, the interpretative value of a biomarker depends on its chemical origin and process context: plant-derived terpenes primarily reflect the biological material and harvest conditions [29,30]; alcohols, acids, and esters describe fermentation when microbial activity is consistent with processing conditions [31]; and pyrazines and furans mainly reflect roasting and precursor composition [35].

## 3. Volatile Compounds in Coffee Harvest

### 3.1. Harvest as a Determinant of Volatile Potential in Coffee

During harvesting, fruit maturity primarily influences the availability of chemical precursors and the integrity of plant tissue rather than the direct presence of aroma compounds characteristic of roasted coffee. At this stage, the concentrations of sugars, amino acids, lipids, and chlorogenic acids establish the chemical foundation that will condition both fermentative development and volatile formation in subsequent processing stages [36]. Consequently, harvest-stage analysis focuses on the evaluation of aromatic precursors and those volatiles detectable in green beans as a result of oxidation, physiological stress, or mechanical damage. In this context, lipid oxidation-derived aldehydes such as hexanal and (E)-2-hexenal, as well as aromatic carbonyls such as benzaldehyde, have been described as early indicators of deterioration or oxygen exposure in green beans [25]. In contrast, compounds such as furfurals and thiols, including 5-methylfurfural and 2-furfurylthiol, are predominantly generated during roasting through thermal reactions, including Maillard chemistry and sugar degradation [23].

When harvesting occurs before reaching this physiological stage, compounds associated with immaturity have been reported, such as 2-furylmethanol or 2-methylpyrazine. These volatiles derive from metabolic pathways active in unripe fruits and are commonly associated with harsh or green flavors in the beverage [37,38]. In green coffee beans, a complex matrix of polysaccharides, lipids, phenolic compounds, and nitrogenous constituents is already present, and its initial composition defines the potential for volatile formation during roasting. For example, hexanal has been identified as one of the main naturally occurring volatile compounds in green Coffea arabica beans, indicating that lipid oxidation or tissue damage may occur prior to any processing [25,39]. Moreover, variability in the composition of different varieties, particularly in the proportion of lipids, sugars, and phenolic compounds, has been associated with differences in the volatiles detected after roasting, suggesting that the initial heterogeneity of the green bean has a direct impact on the final aromatic profile [36].

In contrast, late harvesting favors the accumulation of furans and lactones, such as γ-butyrolactone and 5-methyl-2-furfural, due to the increased availability of sugars and phenolic compounds during over-ripening [40]. This variation demonstrates that the exact timing of harvest modifies the relative proportion of chemical precursors that will later participate in thermal and microbial reactions during postharvest processing. Likewise, the relationship between fruit maturity and the precursors present in the bean influences the formation of subsequent volatiles. The concentrations of trigonelline, caffeine, 5-O-caffeoylquinic acid, and sugars increase as maturation progresses and are considered indicators of the fruit’s physiological development [2]. These compounds are essential because they contribute to the pathways that generate pyrazines, furans, and other volatiles formed during roasting. Their presence at high levels suggests that the bean has accumulated the necessary substrates for greater aromatic complexity in later postharvest stages.

Likewise, studies comparing different harvest times within the same season show variations in pyrazines, ketones, and furans, indicating that environmental conditions prior to harvesting also influence volatile composition [16]. This suggests that the harvesting stage not only determines the immediate volatile profile of the bean but also establishes the chemical foundation that will shape aroma formation during drying and roasting.

### 3.2. Physiological Status and Early Volatile Signatures

Moreover, comparisons between healthy fruits and beans with physiological defects or mechanical damage show that aldehydes such as butanal, benzaldehyde, 3-methylbutanal, and hexanal appear at higher concentrations in defective green coffee beans, in both *C. arabica* and *C. canephora* [37]. These aldehydes originate from accelerated lipid oxidation and may serve as early indicators of fruit deterioration during harvest. Such chemical changes allow the differentiation of healthy and defective beans even before processing, which is relevant because these aldehydes act as early indicators of degradation and directly affect coffee aroma [41].

Chemically, the availability of reducing sugars and free amino acids in mature beans promotes processes in which, during coffee cherry maturation, the cell walls of the mesocarp and mucilage rich in pectins, cellulose, and hemicelluloses undergo softening due to the action of hydrolytic enzymes such as polygalacturonases, pectin methylesterases, and cellulases. This softening and loss of cell-wall integrity has been widely documented as part of the ripening metabolism in coffee fruits [42,43]. The resulting disassembly of the cellular matrix leads to intercellular leakage and a loss of membrane selective permeability, facilitating the release of sugars, organic acids, and amino acids into the surrounding environment [1,44]. Under these conditions, primary alcohols such as ethanol and 1-butanol may be generated through spontaneous fermentations or residual microbial activity, acting as precursors of volatile esters for example, methyl hexanoate and ethyl acetate which impart fruity or sweet notes characteristic of mature fruits [11,45].

In contrast, when fruits exhibit mechanical damage or overripening, lipid susceptibility to oxidation increases, leading to the formation of aldehydes such as hexanal and (E)-2-hexenal, compounds associated with green or herbaceous notes and considered undesirable in high-quality coffees [16,46].

Therefore, controlled maturation enhances the likelihood of controlling the final quality of the beverage, enabling access to more demanding global markets and increasing the probability that producers from different regions receive higher compensation for their beans. For this reason, quantifying compounds in the bean immediately after harvest is a valuable analytical tool for estimating ripeness, damage level, and aroma development. Various studies have shown that aldehydes derived from the enzymatic oxidation of polyunsaturated fatty acids increase significantly in fruits exposed to mechanical stress or oxygen, with hexanal being one of the main indicators of early lipid oxidation [16,17]. Conversely, esters such as methyl phenylacetate and methyl hexanoate are associated with advanced maturation and greater cellular integrity, reflecting a higher availability of alcohols and acids for esterification reactions [47,48]. In this context, these compounds may be considered candidate volatile markers of the harvesting stage, as their abundance is directly influenced by physiological variables (degree of maturity, pericarp integrity) and environmental factors (oxygen exposure, field temperature), which in turn condition the formation of other volatile groups during subsequent fermentation and drying stages [5,49].

Therefore, their early analysis allows the inference of aromatic potential and can guide postharvest strategies aimed at preserving the quality of green coffee. Therefore, the harvesting stage does not merely reflect the physiological status of the fruit but defines the entire aromatic trajectory of the coffee. Fruit maturity regulates the availability of chemical precursors and, consequently, the volatile compounds that will emerge later, including those formed during roasting. Additionally, the physiological condition of the beans leaves detectable chemical signatures such as aldehydes, lactones, or esters that indicate immaturity, overripens, or mechanical damage. These markers provide a biochemical basis for optimizing postharvest strategies aimed at preserving the quality of green coffee.

## 4. Biomarkers in the Coffee Bean Postharvest Stages

### 4.1. Pulping

During the pulping stage of coffee processing, the mechanical removal of the pulp exposes the mucilage to a matrix rich in sugars, pectins, and organic acids which becomes the primary substrate for fermentation. This structural alteration modifies the fruit’s natural microflora and moisture conditions, promoting the activity of yeasts and bacteria present on the bean surface [1,42]. At this point, a metabolic and chemical transition begins, where the increased availability of fermentable sugars and free amino acids stimulates the formation of the first volatile compounds relevant to the final aroma [50].

During pulping, the release of reducing sugars and free amino acids into the mucilage environment supports the activity of fermentative yeasts and lactic and acetic acid bacteria, which metabolize these substrates into short-chain alcohols such as ethanol and 2-phenylethanol [14,18]. From ethanol, acetic acid bacteria can generate acetic acid through oxidation, while the esterification of alcohols and organic acids produces volatile esters such as ethyl acetate and methyl phenylacetate both recognized as early compounds with fruity and floral impact [13,51]. A significant increase in these esters after pulping and initial fermentation has also been reported and is considered a biochemical marker of microbial activity.

In parallel, the release of pectins during pulping promotes the formation of methanol and methyl-pectate oligomers through the action of endogenous pectinases. These products can act as indirect precursors of aldehydes and ketones via oxidative or thermal reactions [5,52]. Likewise, the microbial reduction of sugars generates free glycerol, which can be converted into ketones such as 2-butanone or 2-heptanone compounds frequently detected in coffees processed through rapid pulping. These studies identified more than thirty volatile compounds in early-pulped samples, with esters prevailing over aldehydes in mature fruits, whereas delayed pulping favored aldehydes such as hexanal, associated with oxidation and herbaceous notes [53].

Ethyl acetate and methyl phenylacetate present in the coffee bean can serve as biomarkers of the pulping stage because their formation depends directly on the quality of this operation. Efficient pulping involves the uniform removal of the pulp without causing mechanical damage to the parchment and adequate exposure of the mucilage, enabling the natural release of fermentable substrates primarily soluble sugars, free amino acids, and organic acids. These substrates sustain the activity of a metabolically active microbiota, largely composed of yeasts such as Saccharomyces, Hanseniaspora, and Pichia, along with lactic acid bacteria, which produce alcohols (ethanol, 2-phenylethanol) that are subsequently converted into esters via alcohol-acetyltransferases. For this reason, elevated levels of ethyl acetate and methyl phenylacetate reflect both proper pulping and a balanced initial fermentation, making them useful postharvest biomarkers [14,45]. Conversely, a high proportion of oxidation-derived aldehydes or simple ketones at this stage suggests excessive substrate release or prolonged exposure of the bean to oxygen, conditions that can limit aromatic expression in later processing stages [16,49]. Therefore, this stage leaves chemical signals that define how fermentation will unfold. The way sugars, amino acids, and pectins are released determines whether the microbiota produces esters linked to clean, fruity notes or aldehydes associated with oxidation and green defects. Because these volatiles respond directly to how well the pulping was executed, they offer a practical chemical basis for evaluating the efficiency of the operation.

### 4.2. Fermentation

Postharvest coffee fermentation encompasses a series of biochemical and microbiological processes that occur after pulping and during the degradation of the mucilage surrounding the bean. This stage can be conducted under different systems—natural, honey, washed, or anaerobic/self-induced—each with specific oxygenation, temperature, and microbial conditions that determine the course of metabolic reactions [14,16]. Its primary function is to transform the mucilage and create an environment that promotes the modification of chemical precursors such as sugars, amino acids, and lipids, which are critical for the subsequent formation of volatile compounds in the bean and, ultimately, the aroma of roasted coffee [54,55]. Fermentation may proceed spontaneously through the native fruit microbiota or be controlled via inoculation with selected yeast or bacterial strains. These strategies produce distinct microbial and chemical profiles, reflected in reproducible variations in the coffee’s volatile and sensory profile [5,10].

Microbiologically, coffee fermentation follows a characteristic succession of communities that varies according to the method and environmental conditions. In the initial phases, yeasts primarily *Hanseniaspora*, *Pichia*, *Saccharomyces*, and *Wickerhamomyces* predominate, metabolizing mucilage sugars via glycolysis to produce ethanol, carbon dioxide, and intermediate metabolites, whose main groups and associated volatile compounds are summarized in Table 1 [56,57]. As the process progresses, lactic acid bacteria such as *Leuconostoc* and *Lactobacillus* spp. grow, producing lactic acid, acetoin, and diacetyl, compounds that lower the pH and modify the chemical composition of the mucilage [58,59]. In the later stages, under increased oxygenation and temperature, acetic acid bacteria mainly *Acetobacter* and *Gluconobacter* predominate, oxidizing ethanol to acetic acid, increasing volatile acidity, and generating metabolites that directly influence the sensory quality of the bean [10].

The duration of each phase, which can range from a few hours to several days, depends on variables such as temperature and process management [14]. Recent studies integrating metagenomic and chromatographic analyses have demonstrated clear correlations between the abundance of specific microbial genera and volatile compound formation; for example, *Leuconostoc* and *Hanseniaspora* are associated with the synthesis of aromatic esters and alcohols, while higher Acetobacter activity correlates with increases in volatile acids, primarily acetic acid [10,59].

During coffee bean fermentation, metabolic pathways are activated, explaining the formation of the main volatile compounds at this stage. The catabolism of fermentable sugars through glycolysis produces pyruvate, which under anaerobic conditions is reduced to ethanol by yeast activity [14,18,54]. This ethanol can subsequently be oxidized by acetic acid bacteria to generate acetic acid, a metabolite that increases bean acidity and serves as a marker of aerobic fermentation [11,12,16,55].

Similarly, proteolytic degradation of the mucilage releases free amino acids such as phenylalanine, leucine, and isoleucine, which feed the Ehrlich pathway. In this pathway, amino acids are transaminated and then decarboxylated to produce their corresponding α-keto acids, which are finally reduced to form higher alcohols such as 2-phenylethanol, 3-methylbutanol, and 2-methylbutanol [45,56,57]. These alcohols may be esterified with acetyl-CoA by alcohol acetyltransferases, producing volatile esters such as isoamyl acetate and phenylethyl acetate, which contribute floral and fruity notes typical of well-controlled fermentations [51,59,64,65]. Under certain fermentation conditions, microbial amino acid decarboxylases may also generate volatile amines, such as cadaverine from lysine and putrescine from arginine. These biogenic amines are associated with undesirable aromas and indicate lower quality of the fermented product.

In parallel, lactic acid bacteria convert sugars and organic acids into lactic acid, diacetyl, and acetoin, contributing creamy or buttery sensory notes [56]. Lipolytic and oxidative pathways acting on lipids in the mucilage and pericarp release unsaturated fatty acids, which, through lipoxygenase activity, form aldehydes such as hexanal and 2-hexenal, associated with green or herbaceous notes [34,66]. Yeasts such as *W. anomalus*, *P. kluyveri*, and *H. opuntiae* have been shown to produce high levels of aromatic alcohols and volatile esters due to strong decarboxylase and acetyltransferase activity [16,60]. In particular, the Ehrlich pathway converting phenylalanine to 2-phenylethanol via phenylpyruvate and phenylacetaldehyde serves as a link between mucilage proteolysis and the formation of floral and honey-like aroma compounds, which are considered positive biomarkers of well-controlled coffee fermentations [54,67].

Recent studies quantified an increase in acetic acid during 96-h natural fermentations, from 2.5 mg/g to 6.18 mg/g, whereas pulped or submerged processes, which use the coffee pulp or a liquid-enriched medium for fermentation, showed smaller increases. These changes were accompanied by shifts in the proportion of alcohols and esters and in microbial activity [11,14,18]. In similar studies, glycerol and certain alcohols such as ethanol and 2-phenylethanol were observed to increase during the first 24–72 h, with the ethanol-to-acetic acid ratio varying according to oxygen availability and microbial composition [45,64,65,68]. Collectively, these quantitative data can serve as indicators of fermentation behavior, distinguishing favorable trajectories from those associated with process deviations [12,16,56].

Regarding volatile compounds and their interpretation as potential biomarkers, acetic acid concentrations increase as fermentation progresses due to the growth of acetic acid bacteria. In natural fermentations, acetic acid reaches 4–6 mg/g after 72–96 h, correlating with vinegary flavors and overfermentation [12,14,15]. Conversely, the accumulation of ethanol and acetate esters (e.g., ethyl acetate, isoamyl acetate) is promoted by yeast activity, as yeasts convert sugars into alcohols, which are subsequently esterified by alcohol acetyltransferases [11,45,54]. High levels of these fruity esters (ethyl acetate, isoamyl acetate, ethyl phenylacetate, methyl phenylacetate) correlate with yeast-driven fermentations, associated with fruity and floral notes in the beans [51,68,69]. Overall, the increase in acetic acid versus the accumulation of fruity esters allows clear differentiation of the predominant microbial activity during fermentation: high acetic acid indicates intensified acetic acid bacteria activity and higher risk of overfermentation, whereas dominance of fruity esters reflects yeast-driven fermentation and favorable aromatic profiles. Therefore, the ratio between these compound groups can serve as a useful biomarker for assessing fermentation balance and predicting the sensory quality of coffee beans.

Additionally, 2-phenylethanol and its derivative, phenylethyl acetate, are considered markers of active proteolysis and yeast-driven aromatic metabolism, as their abundance indicates fermentations oriented toward the development of floral or honey-like aromas [14,16,54,57]. On the other hand, acetoin and 2,3-butanediol, compounds of lactic origin, reflect intense lactic activity, contributing creamy or buttery sensory notes, although excessive levels may be associated with sensory defects [14,56]. Finally, aldehydes such as hexanal and 2-hexenal, when persistent at elevated levels during fermentation, reveal lipid oxidation and cellular damage, serving as biomarkers of oxygen exposure or tissue disruption that may lead to undesirable green or herbaceous notes [10,66,70].

Fermentation requires temporal monitoring to track the evolution of physicochemical parameters such as pH and °Brix, alongside microbial counts of yeasts, lactic acid bacteria (LAB), and acetic acid bacteria (AAB), as well as the main volatile compounds formed at each interval (acetic acid, ethanol, 2-phenylethanol, ethyl acetate, hexanal, and acetoin) [11,12,18]. Fermentation not only generates perceptible volatiles but also regulates the amount and balance of key precursors, such as free amino acids and reducing sugars, which will determine the intensity and diversity of Maillard and Strecker reactions during roasting [20,52,71]. These thermal reactions lead to the formation of pyrazines, furans, aldehydes, and other aromatic compounds that define the final character of coffee. Therefore, the interpretation of volatile biomarkers generated during fermentation should be understood as a measure of the bean’s aromatic potential, rather than as a static or isolated profile [1,5,45].

### 4.3. Washing

During this stage, the mucilage surrounding the bean is mechanically removed and rinsed with water to eliminate residual pulp, soluble sugars, organic acids, and metabolites released during fermentation. This procedure, which can involve one or multiple washes, aims to halt microbial fermentation and stabilize the bean prior to drying by modifying the physicochemical conditions of its environment. Water contact and agitation extract soluble compounds, alter moisture content, and reduce the surface microbial load. Depending on the variety, fruit maturity, and method (full wash, partial wash, or soaking), the process affects the composition of the exudate, acidity, and availability of fermentable substrates, directly influencing the synthesis and retention of volatile compounds [51].

Washing acts as a dilution and selective extraction process. The removal of sugars such as glucose and fructose reduces the carbon source for residual yeasts, limiting subsequent production of alcohols and esters. Simultaneously, volatile metabolites generated during fermentation, such as ethanol, ethyl acetate, and phenylethyl acetate, dissolve and are removed with the wash water, altering the bean’s composition [66]. Washed coffees typically exhibit more desirable aromatic profiles, with reduced excessively fermented or vinegary notes, which is attributed to lower free acetic acid levels and the removal of soluble precursors that could fuel later microbial pathways [59].

The magnitude of these changes depends on the timing of the washing step. When washing is carried out immediately after the completion of fermentation, lower concentrations of volatile acids and aldehydes are observed, along with partial retention of fruity esters such as phenylethyl acetate and methyl phenylacetate, which contribute floral and sweet aromas [51]. In contrast, delayed or prolonged washing can remove a significant fraction of these compounds, reducing aromatic complexity and increasing the risk of oxidation. Recent studies report that acetic acid can decrease by up to 30% following a complete wash, while the ethanol-to-ethyl acetate ratio remains stable only in batches washed within 12 h after fermentation [59].

In terms of biomarkers, hexanal has been established as an indicator of oxidative exposure during washing, with increases greater than 20% relative to the fermented bean suggesting excessive air contact or handling. Residual concentrations of esters such as methyl phenylacetate or phenylethyl acetate, even after washing, indicate a balanced fermentation and adequate retention of aromatic compounds within the bean matrix [66]. Likewise, significant reductions in free acetic acid and ethanol can be considered evidence of effective washing and proper fermentation control [69]. Quantitative comparison of hexanal before and after washing allows assessment of the extent to which this stage introduces undesired oxidation, which is critical for preserving the aromatic precursors formed during fermentation. Similarly, the persistence of certain esters coupled with simultaneous reductions in ethanol and acetic acid provides insight into whether washing promotes chemical stability of the bean or, conversely, leads to aroma losses or microbial imbalances [70]. These biomarkers therefore offer a valuable tool for monitoring the technological quality of the washing process.

Thus, the washing process should not be understood merely as a cleaning step, but as a pivotal point in coffee postharvest. Also, the timing and method of washing determine the balance between removal and retention of metabolites, which in turn shapes the chemical profile that will continue to evolve during drying. Improper washing can strip desirable aromas or promote oxidation, while precise control preserves fermentation-derived volatiles and sets the stage for optimal sensory quality [67]. The biomarkers identified at this stage oxidation-related aldehydes, organic acids, and persistent aromatic esters constitute valuable tools for assessing washing efficiency and anticipating the final sensory quality of green coffee [23,69,72]. However, an important knowledge gap remains: systematic scientific studies are still needed to more precisely understand the impact of different types and intensities of washing on the final beverage quality. Should the removal of compounds be maximized at this stage, or is it preferable to allow roasting to modulate and degrade the metabolites accumulated in earlier stages? Addressing these questions is essential for optimizing postharvest practices and developing more rational, evidence-based washing protocols.

### 4.4. Drying

Coffee drying is not only about reducing the bean’s moisture from initial levels of 55–60% to safe storage values of 10–12% but also involves intense physicochemical reorganization of the cellular matrix and the evolution of preformed volatile compounds. As water evaporates, enzymatic and microbial activity decreases, soluble metabolites become concentrated, and non-enzymatic chemical reactions are triggered, many of which are associated with lipid oxidation, sugar degradation, and modifications of free amino acids. The combination of heat, time, and oxygen determines the stability of the bean and the types of volatile compounds that will remain as precursors for roasted coffee aroma [59].

In natural drying methods, typically performed on patios, raised beds, or under shade, moisture loss occurs gradually over several days. In this context, the beans remain in contact with residual mucilage or a thin layer of sugar-rich exudates that, while microbiologically inactive, remain susceptible to slow oxidative reactions. These conditions favor the formation of aldehydes and ketones derived from the oxidation of unsaturated fatty acids, including hexanal, 2-hexenal, and 2-heptanone, produced through the combined action of lipoxygenase and thermal autoxidation. These compounds generally increase progressively until the final stages of drying, with high hexanal levels associated with herbaceous and green sensory notes. Conversely, a gradual decrease in these aldehydes indicates controlled enzymatic deactivation and is linked to gentler and more stable drying processes [34].

Natural drying also favors the retention of aromatic esters and higher alcohols formed in earlier stages. Compounds such as isoamyl acetate, methyl phenylacetate, and 2-phenylethanol can remain within the bean’s protein or lipid matrix due to the slow release of moisture, allowing partial incorporation into the endosperm. These compounds contribute fruity and floral notes, and their persistence has been reported as an indicator of good preservation of aromatic metabolites during natural drying [66]. However, prolonged exposure to sunlight or high temperatures can lead to ester loss through volatilization and increase the formation of free fatty acids, altering the final aromatic balance.

In contrast, mechanical drying is characterized by higher temperatures (35–60 °C) and forced airflow that accelerates dehydration. Under these conditions, chemical reactions intensify and stabilization times are shortened. The combination of heat and oxygen promotes thermal degradation of simple carbohydrates, favoring the formation of furfural, 5-hydroxymethylfurfural, and acetaldehyde compounds that, while contributing sweet or caramel-like aromas at low concentrations, can serve as markers of overheating or excessively rapid drying. Comparative studies in Colombia and Ethiopia showed that mechanically dried coffees exhibited furfural concentrations between 1.5 and 2.3 µg/g, roughly double those of naturally dried beans [51]. Additionally, a significant increase in acetone and 2-butanone, ketones typically generated by thermal degradation of sugars or lipids, was recorded, serving as indicators of processes with higher thermal load [59]. Meanwhile, mechanical drying tends to reduce levels of higher alcohols and esters due to heat-induced volatilization. In particular, concentrations of ethyl acetate and isoamyl acetate can decrease by up to 40% compared to natural drying, while volatile acids such as acetic and butyric acid increase, formed through secondary oxidation of residual alcohols [66]. This shift in the acid–ester balance alters the sensory profile of the green bean, producing drier or more sour notes and reducing fruity complexity.

Drying control not only affects the immediate volatile composition but also influences the chemical stability and aromatic potential of the bean during storage. Excessively rapid drying or temperatures above 50 °C can denature protective enzymes, induce non-enzymatic browning reactions, and degrade free amino acids such as alanine and phenylalanine, reducing the availability of precursors for subsequent Maillard reactions during roasting. In this context, the volatile compounds observed during drying can be considered biomarkers of thermal management and potential coffee quality.

Among the most relevant biomarkers in this stage are hexanal, whose sustained increase indicates active lipid oxidation and thermal damage; furfural and 5-HMF, which reflect heating intensity and the onset of sugar degradation reactions; and residual acetates, whose retention suggests moderate and stable drying. The ratio between furfural and ethyl acetate has been proposed as an indicator of the balance between thermal oxidation and aroma preservation [51]. Thus, a high furfural value accompanied by a low proportion of acetates reflects aggressive drying, whereas the simultaneous presence of both at moderate concentrations indicates controlled drying with good sensory potential.

For both natural and mechanical methods, the drying kinetics should aim for a balance between dehydration and the preservation of internal volatiles. Coffees processed with slow sun-drying maintain more complex profiles of esters and alcohols, while carefully controlled mechanical drying ensures uniformity and reduces the risk of over-fermentation. In both cases, monitoring biomarkers such as hexanal, furfural, acetic acid, and fruity esters, summarized in Table 2, allows for objective evaluation of process quality and prediction of the final aromatic expression of green coffee.

### 4.5. Roasting

During coffee roasting, the peak of aromatic development occurs, resulting from a complex network of thermal reactions that transform the precursors formed and retained throughout postharvest processing [73,74,75,76]. At temperatures ranging from 180 to 240 °C, the bean undergoes dehydration, expansion, and progressive darkening due to Maillard reactions, sugar degradation, and lipid and protein pyrolysis [72,74,75]. These transformations give rise to hundreds of volatile compounds responsible for the characteristic aroma of roasted coffee, including aldehydes, ketones, acids, pyrazines, furans, thiols, and phenolic compounds [77,78]. The composition and abundance of these molecules are closely influenced by the degree of roasting, the rate of temperature increase, and the chemical characteristics inherited from fermentation, washing, and drying [76,79,80].

In the early stages of roasting, when the bean’s internal temperature is still below 160 °C, dehydration and thermal degradation of reducing sugars and organic acids predominate [72]. At this point, aliphatic aldehydes and simple ketones such as acetaldehyde, acetone, and 2,3-butanedione (diacetyl) are released, derived from sugar breakdown via the Strecker degradation pathway [22,47,77,81]. As heating progresses, Maillard reactions between sugars and amino acids generate reactive intermediates such as furfural and 5-hydroxymethylfurfural, which contribute sweet, caramel, and toasted bread notes [82,83]. Simultaneously, decarboxylation and deamination of amino acids produce characteristic Strecker aldehydes, including 3-methylbutanal (from leucine), 2-methylbutanal (from isoleucine), and phenylacetaldehyde (from phenylalanine), compounds associated with cereal, nutty, and honey-like aromas [81].

Between 180 and 210 °C, as the so-called “first crack” approaches, Amadori condensation reactions intensify, and nitrogenous heterocyclic compounds such as pyrazines are formed, which are fundamental to coffee aroma [21,22,74,75]. Methyl- and dimethyl-pyrazines, derived from interactions between sugars and short-chain amino acids (alanine, glycine), impart the toasted, nutty, and malty notes typical of medium-roast coffee [21,75,78]. Recent studies indicate that pyrazines can account for up to 20% of the total volatile fraction in roasted coffee, with the most abundant being 2-methylpyrazine, 2,5-dimethylpyrazine, and 2,3,5-trimethylpyrazine [21,22,76]. These molecules are considered key biomarkers of roast degree, as their concentration increases proportionally with temperature and exposure time, reaching maximum levels in medium roasts and decreasing in dark roasts due to excessive pyrolysis [84].

During the final stage of roasting, above 220 °C, pyrolysis reactions of polysaccharides, lipids, and phenolic compounds predominate [85,86]. The degradation of triglycerides in the bean’s oil generates a variety of medium-chain hydrocarbons and aldehydes, such as nonanal and decanal, as well as ketones like 2-nonanone and 2-decanone, which contribute fatty and creamy notes to the aroma [77,81,87]. Simultaneously, chlorogenic acids, which constitute up to 7–10% of the green bean, break down into caffeic acid, quinic acid, and volatile phenolic compounds such as guaiacol, 4-vinylguaiacol, and cresol, responsible for the smoky, spicy, and phenolic notes characteristic of darker roasts [88,89,90]. These phenolic degradation products serve as thermal indicators of overexposure and are useful as biomarkers of intense roasting [91,92,93].

The formation of furanes and thiols also marks advanced stages of roasting. Furanes (furfural, 2-acetylfuran) originate from the thermal degradation of sugars and sulfur-containing amino acids, particularly cysteine, and contribute sweet, bready, and caramel-like aromas [21,82,94]. 2-Furfurylthiol, in particular, is one of the most characteristic and potent compounds in roasted coffee aroma, described as having notes of freshly ground coffee and intense roast; its formation intensifies between 200 and 230 °C, coinciding with the peak of aromatic development [76,95].

The final profile of roasted coffee depends not only on the generation of these compounds but also on the disappearance or transformation of others that were important during postharvest. Alcohols such as 2-phenylethanol and esters like phenylethyl acetate, abundant after fermentation and drying, are thermally degraded or incorporated into Maillard reactions, acting as precursors for aromatic aldehydes and nitrogenous heterocycles. Thus, the aroma of roasted coffee represents a biochemical continuity from postharvest processes, where each stage contributes substrates that are converted into final volatile compounds [34,75,76,79,80].

Regarding biomarkers, the recent literature has identified several compounds whose presence or proportion allows inference of the degree and quality of roasting. Pyrazines (2-methylpyrazine, 2,5-dimethylpyrazine) are associated with balanced medium roasts and good preservation of nitrogenous precursors [74,76]. Strecker aldehydes, such as 3-methylbutanal and phenylacetaldehyde, indicate an active and controlled Maillard reaction [75,77,81], while an increase in guaiacol and 4-vinylguaiacol suggests a more intense roast or excessive degradation of chlorogenic acids [83,85,89]. Additionally, abundant 2-furfurylthiol and 2-acetylfuran serve as direct indicators of freshly brewed coffee notes and generally correlate positively with sensory scores for medium roasts [59,66,76,95]. Notably, 2-furfurylthiol is a crucial coffee compound, characteristic of the classic aroma of freshly brewed coffee, detectable even in very small amounts. When roasting is performed inadequately either insufficiently (very light roasts) or excessively (very dark roasts) its concentration decreases significantly [35,96].

Variability among coffee varieties also reveals distinctive aromatic signatures. In high-quality Arabica coffees, such as the Geisha variety, comparative analyses using SPME-GC-MS show higher concentrations of limonene and 3-methylbutanoic acid in roasted beans, consistent with their clean, floral, and bright aromatic profile [30,79,97]. Similar results have been observed in comprehensive studies on commercial and wild Arabica varieties, where consistent differences in monoterpenes, volatile organic acids, and phenolic compounds contribute to the unique aromatic profile of each genotype [76,79,98].

In any case, despite the decisive role of key compounds such as thiols, pyrazines, monoterpenes, and furans, it is important to emphasize that the aroma perceived in the final cup results from the synergistic and modulated interaction of multiple molecules, rather than from a single isolated biomarker [75,76,99]. The aromatic expression of coffee is, therefore, an emergent phenomenon arising from the balance between formation, degradation, and presence of volatile compounds throughout postharvest processing, from the green bean to the final brewed beverage [79,99]. Coffee roasting transforms these chemical precursors into a complex array of volatile compounds responsible for the beverage’s characteristic aroma [74,75]. Specific biomarkers such as pyrazines, Strecker aldehydes, and 2-furfurylthiol indicate roast degree, Maillard reaction activity, and sensory quality [76,81,95]. The final aroma emerges from the synergistic interaction of multiple compounds, influenced by roasting conditions and the inherent chemical profile of each coffee variety [79,97]. Proper control of roasting maximizes desirable aromas while preventing the over- or under-development of key volatiles [85,86,87,92].

The usefulness of volatile biomarkers depends not only on which compounds are reported, but also on the comparability of their measurement and the criteria used for their proposal and validation. Therefore, the following section briefly describes some emerging techniques currently applied for the identification of volatile compounds.

## 5. Analytical Approaches for Coffee Volatiles

### 5.1. Headspace–Solid-Phase Microextraction–Gas Chromatography–Mass Spectrometry (HS-SPME-GC-MS)

For initial mapping of compounds across post-harvest stages, headspace-based approaches coupled to GC–MS, particularly HS-SPME-GC–MS, are frequently used because they allow the capture of a broad spectrum of volatile compounds with relatively simple and reproducible sample preparation when the protocol is well controlled. In coffee, this platform has been applied both to describe volatile profiles and to discriminate samples by origin, processing, or roast level, especially when combined with multivariate analysis [100,101]. However, in the context of candidate biomarkers, the critical point is not merely detecting more compounds, but ensuring that observed changes reflect the process rather than the method. In HS-SPME-GC–MS, variables such as fiber type, extraction time and temperature, grinding, humidity, and particularly post-harvest processing conditions can significantly affect the signal. Therefore, this technique is particularly useful for screening when working with matrices in which protocol conditions are reported and transitions between stages are carefully controlled to avoid introducing variability. This is especially important because the stage-specificity required for a biomarker can be lost if uncontrolled methodological variation occurs. HS-SPME-GC–MS is recommended as a comparative method when the matrix is relatively stable or can be standardized. For instance, in green coffee, it is suitable for screening and discrimination by origin or processing when humidity, grinding, and vial mass/volume ratio are controlled, as volatile compounds at this stage are typically low in abundance and sensitive to water-induced partitioning, making humidity standardization essential for comparing batches or treatments. In roasted coffee, the method tends to be more informative and reproducible for aroma fingerprinting because the matrix contains a more abundant and diverse volatile fraction, and headspace release is greater, improving signal-to-noise ratio and discrimination by roast level or aroma profile.

### 5.2. Dynamic Headspace (DHS)

In certain post-harvest situations, target volatiles may be present at very low levels, include highly light fractions, or require a more representative capture of the headspace. In these cases, complementary strategies such as DHS are employed, where volatiles are trapped on sorbent tubes and subsequently released via thermal desorption (TD) for GC–MS analysis. These approaches are considered relevant depending on the matrix and the analytical objective [102], and have been applied in studies aimed at retaining highly volatile compounds or those present at low concentrations [103]. DHS is particularly useful when the sample or process generates a complex headspace that is challenging for HS-SPME, such as when subtle changes are being evaluated in green coffee (early signs of oxidation or storage), when highly volatile profiles are generated during fermentation, or when additional robustness is required for batch comparison. The main advantage of this approach is enhanced detectability and signal consistency for potential candidate biomarkers; however, capture bias depends on the type of sorbent and the sampling strategy, so comparability requires precise reporting of sorbent type, sampling time, flow rate, and blank controls. When volatile compounds are present at very low concentrations or dominated by highly volatile fractions, DHS is the method of choice. Its primary benefit is increased detectability and stability of the signal by concentrating the headspace and transferring it via thermal desorption to the GC–MS. Nevertheless, selectivity and potential bias depend on the sorbent and sampling regimen, making rigorous reporting and standardization essential for reliable comparison.

### 5.3. Solvent-Assisted Flavor Evaporation (SAFE)

In studies aimed at isolating aroma fractions with reduced matrix interference, methods such as SAFE (Solvent-Assisted Flavor Evaporation) or simultaneous distillation–extraction are employed. These approaches recover compounds that may not be well represented in the headspace due to low volatility or retention within the matrix. Therefore, they are particularly suitable for green coffee and post-harvest matrices with high water and solid content, where headspace sampling may not consistently capture compounds of interest, and also when confirmation of compounds associated with quality or defects is required through a broader extracted fraction. Their contribution to biomarker studies is complementary, primarily providing interpretative support; however, because the solvent and extraction conditions can bias the profile, they are not recommended for direct comparisons between stages or between studies unless the procedure is strictly standardized [104].

### 5.4. Gas Chromatography–Olfactometry (GC–O)

When a candidate biomarker is proposed as an indicator of sensory quality, it is not enough for the compound to be merely present or statistically significant; it must also have olfactory relevance. In coffee, this is addressed using GC–O combined with aroma dilution analysis and by considering the relationship between compound concentration and its odor threshold. This approach allows the differentiation between compounds that are simply detectable and those that truly determine aroma. It is particularly relevant for compound families known to contribute to sensory attributes, such as sulfur compounds with high odor potency, pyrazines associated with roasted or nutty notes, certain volatile phenols linked to smoky or spicy profiles, and furanoids contributing caramelized notes. Interpretation, however, depends on the processing stage. In commercial coffee, GC–O has been used to explain sensory differences between batches and to support decisions regarding profile standardization [24], and its methodological basis is extensively discussed in reference texts on sensory evaluation and food chemistry [105]. This approach is therefore most applicable to roasted coffee and prepared beverages or extracts, where the final aroma is fully expressed and the olfactory contribution of compounds can be assessed directly and reproducibly.

### 5.5. Comprehensive Two-Dimensional Gas Chromatography–Time-of-Flight Mass Spectrometry (GC×GC–TOF-MS)

In matrices where dense patterns require higher separation, high-capacity approaches such as GC×GC–TOF-MS are employed, often integrated with chemometric analysis to discriminate signals associated with variety, origin, processing, or roast level. This strategy has been applied in coffee when the volatile profile is complex and greater separation and classification power are needed [106,107]. The analytical technique reduces false markers arising from co-elution in conventional GC and allows the construction of more robust signal sets when the goal is discrimination by stage or origin. Therefore, it is most suitable for roasted coffee and beverage evaluations, where the diversity and density of compounds increase the risk of overlap and a higher separation capacity is required to discriminate by roast, origin, or processing.

### 5.6. Proton-Transfer-Reaction Time-of-Flight Mass Spectrometry (PTR-ToF-MS)

During roasting, chemical changes occur on very short time scales; therefore, spot sampling with conventional GC may not adequately capture the kinetics of compound formation and release. In this context, online or high-temporal-resolution techniques such as PTR-ToF-MS allow near real-time monitoring of changes and the correlation of operational parameters (time/temperature) with measurable chemical signals. This is particularly useful when volatiles are intended to be used as control variables for adjusting the roasting process [108,109].

### 5.7. Gas Chromatography–Ion Mobility Spectrometry (GC–IMS)

Finally, when the objective is rapid classification or the construction of aroma fingerprints for discrimination, tools such as GC–IMS and electronic nose (e-nose) sensors, often integrated with chemometrics or machine learning, have been explored [76]. In the context of biomarkers, these tools mainly serve as discrimination or alert systems; their contribution is greatest when used as an initial classification layer, supported by confirmatory methods such as GC–MS or GC–O for chemical interpretation. Together, these methodologies address distinct analytical needs: candidate discovery, confirmation and comparability, sensory relevance, resolution in complex matrices, monitoring of roasting dynamics, and rapid classification. This structured approach enables the discussion of volatile biomarkers based on their intended use and evidence, minimizing the risk that instrumental differences are mistaken for stage- or quality-related signals.

## 6. Future Perspectives

### 6.1. Biomarkers as Indicators of Cup Quality

The association between biomarkers and cup quality is strengthened when a marker is interpreted as a signal that traverses the post-harvest flow, rather than as a compound specific to a single stage. The goal is not merely to identify what appears during harvest, fermentation, or roasting, but to determine which signals are generated early, which are attenuated or amplified by subsequent handling, and which ultimately manifest in measurable attributes during cupping. This requires working with signals that act as early indicators of risk and signals that function as positive attributes, while evaluating how they interact throughout processing.

During harvest, initial conditions are set that are difficult to reverse. If coffee enters processing with mechanical damage, prolonged oxygen exposure, or extended time before processing, its composition shifts toward compounds associated with deterioration. This deviation can persist through drying, where it is quantified as a loss of stability and an increase in oxidative markers. In green coffee, such signals are not interpreted as aroma, but rather as the probability that the lot will reach roasting with reduced capacity to achieve high cup scores. Evidence distinguishing origin, post-harvest processing, and roast level shows that the relevance of a signal can change depending on the sample state, highlighting the role of early biomarkers in anticipating risk rather than indicating positive attributes [34,49].

After pulping and during fermentation, the scenario changes, as the chemical signal is no longer dominated by deterioration but depends on microbial pathways and operational control. When studies report composition and cupping results under defined conditions, it becomes possible to observe which chemical changes accompany improvements or losses in sensory attributes, identifying signals meaningful as applied biomarkers. Fermentation interventions have been shown to induce simultaneous changes in volatile and non-volatile compounds as well as in sensory outcomes, and fermentation duration has been linked to differences in total score and descriptor performance. This allows the identification of candidate signals that are not only differential but also consistent with process behavior [20,110,111].

A key point in linking biomarkers to cup quality is quantifying how much of the signal is preserved during drying, a stage where poor handling can erase or distort information generated during fermentation. This is not because a compound disappears in isolation, but because the balance of compounds shifts and undesired compounds are formed, competing sensorially and degrading attributes such as cleanliness and sweetness. Therefore, the most valuable biomarkers for cup quality are those that maintain consistency as the lot progresses under variable physical conditions rather than appearing in a single stage. From this perspective, methodological advancement involves using cupping attributes as response variables and chemical profiles as predictors in multivariate or supervised models, reporting performance and validation under real variability. Chemical fingerprints combined with machine learning capture structured information useful for classification, and rapid detection tools such as electronic noses can function as an alert layer, with subsequent confirmation by chromatographic techniques when chemical interpretability is required [112,113,114].

### 6.2. Validation of Coffee Volatile Biomarkers

The main barrier to establishing volatile biomarkers in post-harvest coffee is the lack of evidence regarding their performance under real-world conditions and with comparable protocols. Therefore, the focus should shift from simply reporting candidates to defining minimum requirements for measurement, quality control, and validation. The first critical point is sampling: differences have been observed between individual beans within the same roasted sample, which necessitates establishing homogenization strategies, sample size, and number of replicates before attributing effects to stage or quality [6]. In the field, humidity and temperature must also be recorded or controlled, as they affect volatile release and partitioning, potentially altering readings even if the process itself has not changed.

Moreover, standardization of methods on platforms such as HS-SPME-GC-MS, including the use of different fibers and extraction, grinding, and moisture conditions, affects relative intensities. Therefore, comparability across studies requires detailed reporting of the protocol and analytical acceptance criteria [101,115]. Additionally, fingerprint-based classification functions under controlled conditions and require explicit strategies to account for differences in analytical instruments, equipment variation, and environmental conditions. Several reviews in food analysis discuss standardization frameworks and performance criteria to ensure consistent decision-making in quality control and authenticity [116,117,118]. In electronic noses, sensor drift and the need for recalibration are major limitations, confining their use primarily to alert or classification purposes, with confirmatory analysis required for precise chemical identification [119,120].

In this context, the use of machine learning is feasible if integrated with validation. Supervised models can handle multicomponent fingerprints and reduce reliance on a single marker, but they must be trained with real variability, include external validation, report metrics, and document updates or recalibration when the measurement environment changes. A practical approach is validation in stages: first, repeatability in the laboratory; next, performance under variability representative of production; and finally, integration with cupping when the objective is final quality. Evidence from real farm conditions indicates that the field component is necessary to build transferable models [121]. In the short term, it is more realistic to deploy reduced panels or fingerprints at critical points in the process, combining rapid screening techniques with chromatographic methods for confirmation when necessary [116,122].

## 7. Conclusions

Overall, the collected evidence indicates that the volatile profile of coffee is shaped by the interaction of biosynthetic and abiotic pathways throughout post-harvest processing. Therefore, a single compound or chemical family should not be interpreted as a stage-specific marker without considering its dominant mechanism and the conditions of the process. This distinction is critical to avoid misattributions and to select biomarkers with operational relevance. The next step in the field is not to expand lists of compounds, but to transform candidates into transferable tools. This requires defined reference ranges and decision thresholds by origin and processing system, as chemical responses are not universal across varieties, regions, or post-harvest practices. Concurrently, the chemical–cup relationship should be formalized through qualitative or quantitative models that link specific compounds or combinations of families with professional cupping sheet indicators, including cleanliness, sweetness, flavor, aftertaste, and defects, with external validation and reported performance.

Transfer to real-world conditions requires comparable protocols and in situ validation. Study designs should incorporate lot heterogeneity, the effects of moisture and temperature, and data quality control to separate process-driven changes from analytical variation. In this framework, it is more realistic to apply reduced panels or aroma fingerprints at critical points in the post-harvest flow rather than rely on a single universal biomarker. Finally, technological development should prioritize rapid measurement for classification and alert purposes in production, with chromatographic confirmation when chemical traceability is required, and online monitoring during roasting to link volatile signals with real-time control of the thermal profile. This approach integrates mechanism, validation, and application, converting volatile compound analysis into a process control tool oriented toward sensory quality.

## Figures and Tables

**Table 1:** Volatile compounds produced by microorganisms during coffee bean fermentation.

Microorganism	Group	Reported Volatiles	Aroma/Descriptor	References
*Saccharomyces cerevisiae*	Yeast	Ethanol; ethyl acetate; isoamyl acetate; 2-phenylethanol	Fruity, floral, sweet (esters and higher alcohols)	[46]
*Hanseniaspora* spp. (*H. uvarum*, *H. opuntiae*)	Yeast	Ethyl acetate; phenylethyl acetate; aromatic alcohols	Fruity, floral; contributes to fruity esters in early fermentation	[58]
*Pichia*/*Issatchenkia* (*P. kudriavzevii*)	Yeast	Ethanol; ethyl acetate; isoamyl acetate; 2-phenylethanol	Fruity, alcoholic; enhances fruity notes	[60]
*Wickerhamomyces anomalus*	Yeast	2-phenylethanol; acetates; various alcohols	Floral, fruity; increases aromatic compounds in inoculation trials	[61]
*Kluyveromyces*/*Kazachstania* (*K. marxianus*)	Yeast	Higher alcohols, esters	Fruity, floral	[62]
*Weissella*/*Leuconostoc/Lactobacillus* (*LAB*)	Lactic acid bacteria	Diacetyl (2,3-butanedione), acetoin (3-hydroxy-2-butanone), ethyl lactate	Creamy, buttery, milky notes; can modulate acidity and produce ester precursors	[57]
*Acetobacter*/*Gluconobacter* (*AAB*)	Acetic acid bacteria	Acetic acid; ethyl acetate; other acetates	Acidic/vinegary if excessive; moderate levels add fruity complexity	[63]
*Bacillus* spp.	Bacilli	2,3-butanedione (diacetyl), 2,3-butanediol, nitrogenous compounds	Creamy, buttery, earthy (depending on species)	[58]
*Filamentous*/*Environmental Fungi* (*Geotrichum*, *Mucor*, *Penicillium*)	Fungi	Aldehydes, alcohols, rancid compounds if proliferating	Green, earthy, rancid (indicative of contamination or poor handling)	[61]

**Table 2:** Volatile Compounds at Each Postharvest Stage of the Coffee Bean.

Stage	Predominant Volatile Compounds	Chemical/Biochemical Origin	Sensory Descriptor	Interpretation as Biomarker	References
Harvesting	2-methylbutanal, 4-ethylguaiacol, butanal, benzaldehyde, 3-methylbutanal	Lipid oxidation and tissue damage associated with fruit maturity, mechanical stress, and oxygen exposure	Green, fresh, herbaceous	Early indicators related to fruit integrity and oxidative status of green coffee beans; potential candidate markers associated with harvesting conditions	[1,25,34]
Pulping	Hexanol, 2-heptanol, octanal	Enzymatic oxidation post-cell rupture	Fruity, green	Marks the onset of microbial and oxidative activity	[51]
Fermentation	2-phenylethanol, ethyl acetate, phenylethyl acetate, acetic acid	Alcoholic and lactic acid fermentation (yeasts and LAB)	Floral, fruity, acidic	Indicators of controlled fermentation and aromatic quality	[54,59,70]
Washing	Medium-chain aldehydes, aliphatic alcohols	Mild oxidation of residual sugars and lipids	Clean, fresh	Evidence of transition toward dehydration	[59]
Drying	Hexanal, 2-octenal, 2-pentylfuran, butyric acid	Oxidation of lipids and phenolic compounds	Fatty, cereal-like, rancid (if excessive)	Biomarkers of green coffee oxidative stability	[5,17]
Roasting	2-methylpyrazine, 2,5-dimethylpyrazine, 5-methylfurfural, furfural, guaiacol, 2-furfurylthiol	Maillard, Strecker degradation, pyrolysis	Roasted, nutty, caramel, coffee	Indicators of roast degree and final aromatic development	[6,23,27]

## Data Availability

No new data were created or analyzed in this study. Data sharing is not applicable to this article.
